# What makes us conscious of our own agency? And why the conscious versus unconscious representation distinction matters

**DOI:** 10.3389/fnhum.2014.00434

**Published:** 2014-06-23

**Authors:** Glenn Carruthers

**Affiliations:** ARC Centre of Excellence in Cognition and Its Disorders, Macquarie UniversitySydney, NSW, Australia

**Keywords:** consciousness, self-consciousness, sense of agency (SoA), hard question, functionalism, vehicle theory

## Abstract

Existing accounts of the sense of agency tend to focus on the proximal causal history of the feeling. That is, they explain the sense of agency by describing the cognitive mechanism that causes the sense of agency to be elicited. However, it is possible to elicit an unconscious representation of one’s own agency that plays a different role in a cognitive system. I use the “occasionality problem” to suggest that taking this distinction seriously has potential theoretical pay-offs for this reason. We are faced, then, with a need to distinguish instances of the representation of one’s own agency in which the subject is aware of their sense of own agency from instances in which they are not. This corresponds to a specific instance of what Dennett calls the “Hard Question”: once the representation is elicited, *then what happens?* In other words, how is a representation of one’s own agency used in a cognitive system when the subject is aware of it? How is this different from when the representation of own agency remains unconscious? This phrasing suggests a Functionalist answer to the Hard Question. I consider two single function hypotheses. First, perhaps the representation of own agency enters into the mechanisms of attention. This seems unlikely as, in general, attention is insufficient for awareness. Second, perhaps, a subject is aware of their sense of agency when it is available for verbal report. However, this seems inconsistent with evidence of a sense of agency in the great apes. Although these two single function views seem like dead ends, multifunction hypotheses such as the global workspace theory remain live options which we should consider. I close by considering a non-functionalist answer to the Hard Question: perhaps it is not a difference in the use to which the representation is put, but a difference in the nature of the representation itself. When it comes to the sense of agency, the Hard Question remains, but there are alternatives open to us.

## Introduction

In this paper I argue that we, as a community investigating the sense of agency, are not doing enough to answer what Dennett has called the “Hard Question” of consciousness. Our existing models do a very good job of explaining when a representation of own agency is elicited. I illustrate this with two historically important accounts: the comparator model of Frith et al. and Wegner et al. inference to apparent mental state causation. Following Revonsuo, I consider these to be proximal etiological explanations. Although powerful so far as they go, these accounts, on their own, do not provide us with the explanatory resources to distinguish conscious and unconscious representations of one’s own agency. This is not a problem we can ignore. I use the “occasionality problem” to suggest that there are potential theoretical benefits to taking this distinction more seriously as conscious and unconscious representations of own agency play very different roles in cognition. I conclude by considering how we might approach the Hard Question for the sense of agency. I consider two Functionalist approaches (i) that a representation of own agency is conscious if it is taken as the object of attention; and (ii) that a representation of own agency is conscious if it is available for verbal report. Although such approaches offer clear research agendas, both of these specific approaches seem non-starters on empirical grounds. That said multifunction hypotheses such as the global workspace theory remain viable Functionalist positions. Finally I consider a Vehicle theory approach to the Hard Question. Such an approach also offers some clear research questions, but currently no clear answers. As of now, the Hard Question remains under-considered for the sense of agency even though there exist a variety of questions we can ask to make progress on it if we take either a Functionalist or a Vehicle approach. These are questions we would all do well to consider.

## Standard accounts of the sense of agency

Standard explanations of the sense of agency are of a particular type. Revonsuo (2006, pp. 20–22) calls this type of explanation “proximal etiological explanation”. Such explanations have two defining characteristics. First, they enumerate *causes* of the sense of agency. Second, the explanations are *cognitive* explanations. The specific causes posited are mental representations and computations. To understand these accounts as explanations of the sense of agency then, is to understand them as a description of what aspects of the mind, i.e., mental representations and computations, cause a subject to experience their own agency. The sense of agency itself is taken to be just another representation in this causal chain.

These traits are shared by prominent accounts of the sense of agency. Consider first the comparator model. This model gets its name from the use of three hypothetical comparisons performed by the motor control system. Each of these comparisons performs specific functions for motor control and motor learning (Wolpert and Ghahramani, 2000). One of these comparisons also elicits the sense of agency. This is the comparison that will concern us here (for the full account and its broader applicability see Frith et al., 2000a). On this model it is hypothesized that performing an action requires the formation of a goal state or motor intention (Pacherie, 2008), which represents where the body needs to move to in order to perform the action. From this, the motor control system formulates a motor command, which specifies how to move the body from where it is to where it needs to be in order to attain the goal. Two copies of the motor command are formed; one is sent to the periphery and elicits the requisite contractions of the effector muscles to perform the movement needed to attempt the action. This movement, of course, affects the sensory organs, allowing the motor control system to represent the movement after it occurs. The second copy of the motor command, sometimes called the “efference copy” or “corollary discharge”, is used by the motor system to form a prediction of what sensory feedback will be received due to the action. This predicted feedback can be used to represent the action as it occurs (Frith et al., 2000a,b; Blakemore et al., 2002).

Now we get to the sense of agency. It is hypothesized that the collection of representations and computations introduced above cause the sense of agency to be elicited. Specifically when feedback from the senses to the motor control system (actual sensory feedback), matches an internally generated prediction of what this feedback will be (predicted sensory feedback), a sense of agency is elicited (Frith et al., 2000a, p. 1784). These two representations matching in this context means that they represent the same action. The comparator model has been considered a promising explanation of the sense of agency and is able to explain some important discoveries (for recent reviews of what and how see Carruthers, 2012).

Wegner et al. have suggested that the sense of agency is elicited by a rather different kind of computation. On their model, the sense of agency is elicited when one infers that one or other of one’s mental states caused the action of one’s body (Wegner and Wheatley, 1999, p. 480; Wegner, 2003, p. 67). If correct, we can provide a proximal etiological explanation of the sense of agency by explaining how this inference is made. To make the inference the subject needs to represent their mental states *qua* potential causes of action, represent which body in the world is their own (i.e., a sense of embodiment) and represent the action which is occurring or has occurred. Next they must represent that one or other mental state causes the action of their body. This is the role of the inference to apparent mental state causation. According to Wegner et al. this inference is made when three facts about the relationship between the mental state and action are recognized. First, the mental state must be consistent with the action in that it specifies the action that actually occurs. Second, the mental state must seem to occur at an appropriate time before the action occurs, for example a memory of an action won’t be inferred as a cause of that action. Third the thought must appear to be the only possible cause of the action, i.e., if something else, another person or gust of wind, say, could have caused the action then the inference will not be made, or at least not made with a high degree of certainty. Wegner et al. call these the principles of “consistency”, “priority” and “exclusivity” respectively (Wegner and Wheatley, 1999, pp. 482–487; Wegner, 2003, p. 67). Like the comparator model, this account has been considered a promising approach to the sense of agency and it can explain some important discoveries (see Wegner, 2002 for reviews; Carruthers, 2010).

Numerous authors have followed the general approach of these classic hypotheses. Recently, several authors have proposed that the sense of agency is elicited by a process that integrates the output of several such computations (Synofzik et al., 2008, 2009; Moore and Fletcher, 2012; Carruthers, in press). This work is characterized by considerable progress in investigations into the computations that elicit the sense of agency. However, there is a limitation to this approach. Knowing how the representation of agency is elicited doesn’t distinguish between cases where it is elicited but remains unconscious and cases where it is elicited and the subject is aware of it. Unless we are to view the sense of agency as unique amongst all mental representations in that it can only *ever* be conscious, we must allow for the possibility of a representation of own agency to be elicited and remain unconscious. In the next section I consider the occasionality problem which has been presented as an objection to the comparator model, but which is generalizable to other accounts. I use this as an example to show that taking seriously the distinction between unconscious representations and a conscious sense of agency can have theoretical pay-offs in this area as each of these play different roles in the broader cognitive system. In particular I suggest that if we take this distinction seriously then the occasionality problem doesn’t arise.

## The occasionality problem and unconscious representations of one’s own agency

It would, I take it, be bizarre if there were no unconscious representations of own agency. But, is there any theoretical benefit for sense of agency research as it it currently done to considering this fact explicitly? In this section I argue that there is. That by considering the different roles conscious and unconscious representations of own agency play, in particular that only the absence conscious representations can be noticed by the subject, we can avoid the “occasionality problem”. In the next section I consider some ways in which we might attempt to explain the difference between conscious and unconscious representations of own agency. The occasionality problem, it should be noted, was originally formulated as an objection to the comparator model, but it applies equally to Wegner et al. account described above. To see the problem we first need to take a step back and consider clinical phenomena that the above models need to explain.

One of the central explananda for the accounts introduced above is thought to be delusions of alien control. This delusion, commonly seen as a symptom of schizophrenia, is a patient’s belief that not they, but rather some other agent, control the patient’s actions. This is expressed in reports such as:

I felt like an automaton, guided by a female spirit who had entered me during it [an arm movement].

I thought you [the experimenter] were varying the movements with your thoughts.

I could feel God guiding me [during an arm movement] (Spence, 2001, p. 165).

There is a growing consensus that explanations focusing on the sense of agency alone cannot explain every feature of this delusion (Synofzik et al., 2008; Carruthers, 2009). In particular, such accounts do not have the resources to explain why patients attribute the action to another specific agent. What such accounts can explain is why patients fail to attribute their actions to themselves. According to the comparator model, in healthy subjects the comparison between actual and predicted sensory feedback causes a sense of agency to be elicited for actions the subject performs. However, it is hypothesized that this computation goes wrong for the patient suffering delusions of alien control. They do not represent a match between predicted and actual sensory feedback when they should and so no sense of agency is elicited. Without this sense, the patient has no experiential basis for a self-attribution of action- they do not feel as though they perform the action- and so actions are not self-attributed. For those interested in the details of why this occurs, there is some experimental evidence that these patients have an underlying deficit in forming or using predicted sensory feedback (Frith and Done, 1989; Blakemore et al., 2000; Carruthers, 2012).

As with the comparator model, Wegner et al. inference to apparent mental state causation is unlikely to explain every feature of this delusion. Like the comparator model it may offer an account of how the sense of agency is lost. According to this view the sense of agency would be lost when one of the principles of priority, exclusivity or consistency is not met. I have argued elsewhere (forthcoming) that on this model it is reasonable to hypothesize that it is the principle of priority which is violated, as there is some evidence that patients suffering from delusions of alien control display abnormalities in the representation of the timing of their actions (Voss et al., 2010).

Now we are in a position to examine the occasionality problem (de Vignemont and Fourneret, 2004; Proust, 2006, p. 89; Synofzik et al., 2008). This problem starts from the observation that those suffering from delusions of alien control only attribute *some* of their actions to other agents. None of the models above appear to have, on their own, the resources to explain this observation. At the core of this objection is an accusation of a false prediction. A model like the comparator model predicts patients lack a sense of agency for their actions because they cannot represent a match at the comparison between actual and predicted sensory feedback. This does not offer us principled grounds for distinguishing those actions that the patient self-attributes and those that they attribute to others. If the comparison fails then the model should predict that patients lack a sense of agency for all of their actions. This is not the case, so the comparator model appears to be incorrect. This problem arises again when we consider Wegner et al. account. Hypothesizing that these patients fail to represent their own agency for their actions because they misrepresent the timing of their actions (thus violating the principle of priority) again fails to explain why only some actions are misattributed. In essence these accounts suggest that such patients always lack a representation of their own agency, but it seems that this lack only matters to the subject some of the time.

de Vignemont and Fourneret, (2004, p. 9) have suggested that the system which elicits the sense of agency, whether it be the comparator model or something else, fails only occasionally and in a context specific way. If it is true that the comparator or inference to apparent mental state causation face intermittent failures, then the occasionality problem disappears. However, the questions of how and why the mechanism occasionally fails have not been answered and nothing about the actions themselves or features such as their personal significance affect whether or not they are self-attributed (Proust, 2006, p. 89). More importantly there is no evidence independent of reports of the delusion that the comparator model or the representation of the timing of actions fails only occasionally for such patients. Until such evidence is forthcoming it is difficult for this solution to shake the appearance of being *ad hoc* and it is worth considering other accounts. More so, as I will suggest below, if we consider the different roles conscious and unconscious representations of own agency play in cognition, which we should do anyway, then there is no need to add additional assumptions of this type.

An argument from the occasionality problem against the hypotheses described, like that sketched above, assumes that the result of the process leading to a representation of own agency is a conscious sense of agency. If we drop this assumption the problem needn’t arise. To see why this assumption is being made let us consider the relationship between experiences and delusions. So, what is the evidence that patients suffering delusions of alien control lack a sense of agency? One might be tempted to think that they *say so*. But, this isn’t typically the case. Rather a deficit in a conscious sense of agency is inferred from the fact that patients attribute their own actions to another agent. This inference is justified by some standard assumptions in the study of delusions. The state of the art in delusions research is strongly influenced by Maher (1988, 1974) hypothesis that delusions are attempts to explain anomalous experience. Now there may be controversy regarding whether this explanatory attempt involves normal or deficient reasoning (Davies et al., 2002; Gerrans, 2002), but both sides agree that the delusion arises from an attempt to make sense of an anomalous experience. This supposition is not universally accepted, of course (Campbell, 2001; Bayne and Pacherie, 2004), but what matters here is that this assumption is needed if we are to justify inferring that patients lack a sense of agency from their acts of other attribution. We can justify this inference if the lack of a sense of agency is the anomalous experience which the delusion of alien control is an attempt to make sense of. So first, why should the absence of a sense of agency be an anomaly that needs to be explained? Well, it would be, if a conscious sense of agency typically accompanied one’s actions. If this is the case, we would expect that its absence would be noticed and felt to be in need of explanation. After all, if one feels one’s body move, but one does not seem to be the agent behind the movement, then one would naturally search for a reason that one moved.

We see this assumption that there is a conscious sense of agency accompanying all actions at play in the argument from the occasionality problem. The general failure of a process like the comparator should mean that the sense of agency that is usually present is not. This is an anomaly to be explained by the patient. The patient should show delusions of alien control for all of their actions, but they do not, therefore the comparator model (or which ever process we are considering) is false, quod erat demonstrandum (QED).

To avoid this conclusion, all we need do is drop the assumption that a conscious sense of agency always accompanies our actions. Instead, we need only hypothesize that a representation of own agency which may or may not be conscious accompanies our actions. In other words, the output of processes like the comparator or inference to apparent mental state causation is a representation of the subject’s agency which is sometimes conscious and sometimes not. An absence of a sense of agency is thus not always an anomaly which the patient need explain. An absence of representation is not a representation of absence, as the saying goes, and it is particularly not a representation of absence *to the subject*. It is the subject noticing (i.e., representing to themselves) that the sense of agency is absent which is hypothesized to lead to delusions of alien control, not it’s mere absence. This noticing of the absence will occur when the sense of agency is expected and so we might say the absence of a sense of agency is only an anomaly when the subject expects to experience it.

A possible objection to this line of response is to assert, based on introspection, that a conscious sense of agency accompanies all of our actions in the normal case. As such, it is always expected and any absence is an anomaly to be explained. However, introspection gives us poor grounds to assume that there is a ubiquitous sense of agency. What would lead one to assume that there is a conscious sense of agency accompanying every action? We can see where this assumption comes from, and how poorly grounded it is, by an analogy with visual consciousness. A favorite example purporting to show that we are not conscious of as much as we think we are comes from Dennett (1991). This example is so easy to replicate that given minimal resources you can do it yourself right now. All you need is a well shuffled deck of playing cards. Stare at a point on a wall in front of you. It is important that you continue to stare at this point throughout the entire demonstration. Without looking randomly select a card and hold it out to one side at arm’s length. Gradually move it toward the center of your vision. At what point can you see the color and number on the card? The typical finding is that it is only about 2 or 3°^1^ from the point one is looking at that these features become visible (Dennett, 1991, p. 54). The reason for this is to do with the nature of photoreceptors outside of the fovea on the retina and need not concern us here. What I wish to draw attention to, however, is that on first experiencing this demonstration most people seem surprised (Dennett, 1991, p. 68). Pre-theoretically, we expect to be able to discriminate objects easily when they are presented in our peripheral vision. Dennett suggests, and I agree, that this expectation is based on a folk-theoretical belief that vision presents us with a relatively uniformly clear and colored world in which objects are easily distinguished. But, as this simple demonstration shows, as do other more rigorous experiments, e.g., Brooks et al. (1980), this is at best only true of the foveated world, and even then with some exceptions (Caplovitz et al., 2008).^2^

Why do we believe this is true of our peripheral vision? We can speculate on many possible reasons for this. One reason might be that things we use as public representations of what we see, e.g., photographs or videos, are somewhat like this. There may be a misbegotten analogy between visual depictions and visual experience. Another more universal proposal comes from Schwitzgebel (2008: p. 255) as well as Dennett (1991: p. 68) who suggests that objects in our peripheral vision appear distinct and colored because they are when we look at them. Whenever one looks to see what object is in one’s periphery one finds it clear, distinct and colored. As such we tend to assume that we always experience those objects as such. This claim provides us with a useful analogy for understanding why accounts like the comparator model and Wegner et al. inference to apparent mental state causation needn’t suppose that a conscious sense of agency accompanies every action.

If a model like one of those above is right, then it would be true that our actions are normally accompanied by a representation of our own agency. However, the subject need not be aware of their own agency. The representation could be unconscious but, because the representation is formed with every action, it is there whenever we go “looking” for it, or more generally when it is expected to occur to the subject, i.e., consciously. Just as objects in our periphery always appear clear, distinct and colored when we go looking for them, our representation of agency is always experienced when we go looking for it, thus meeting our expectations. Just as this may lead us to believe that objects in our periphery always appear clear, distinct and colored, this may lead us to believe we always experience a sense of agency accompanying our actions rather than merely representing it.

Accepting this conclusion then, the comparator model or the inference to apparent mental state causation need not suppose that representing one’s own agency is always a conscious sense of agency. Still, one may wonder, how exactly does this affect the occasionality problem? After all, it would still seem to be the case that these models predict that the unconscious representation of agency would be missing for every action performed by the patient suffering delusions of alien control, so should the model still predict that the patient would show the delusion for every action?

The answer to this is no. However, to see why, we need to return to the purported role of consciousness in the formation of delusions such as delusions of alien control. Recall Maher’s proposal that delusions are attempts to make sense of anomalous experiences. In the case we are interested in here, the delusion of alien control arises because the patient attempts to make sense of the absence of a sense of agency. They expect a sense of agency, but it is not there when they “look”, giving rise to an anomalous experience that must be explained. On this view then, an absent sense of agency is only anomalous when it is expected. A subject would not notice the absence of an unconscious representation. It is only when the representation would otherwise become conscious that its absence would be noticeable. Again the absence of the representation is not the same as the subject representing to themselves that something is absent. The upshot of this is that if we hypothesize that the comparator or inference produces an unconscious representation of agency, which only becomes conscious when it is needed by the subject (say in self-recognition or introspecting to see what experiences one has), we find that the occasionality problem is no problem at all.

It is not so much that the problem is solved as it doesn’t arise in the first place, all because conscious and unconscious representations of own agency play different roles in cognition. Only conscious representations can be expected by the subject, and only their absence can be noticed by the subject. The normal case is that actions are not accompanied by a conscious sense of agency (only an unconscious representation) and so a lack of this feeling is typically not an anomaly that the patient suffering delusions of control needs to explain. It is only when they would “look for” (however this analogy is to be cashed out mechanistically—see below) this representation that it is expected and so its absence is an anomaly that needs to be explained.

This consideration of the occasionality problem shows us that there are theoretical benefits to taking seriously the distinction between conscious and unconscious representations of own agency. By doing so and considering the different roles conscious and unconscious representations play in cognition we see that the occasionality problem doesn’t arise. As such we don’t need to add assumptions to our models, such as assuming that they only fail some of the time, which lack supporting evidence. However, we do have a new set of issues to consider. What then is the analogy of “looking for” the representation of agency that produces the expectation of the sense of agency needed to explain delusions of alien control? This question is no less than what distinguishes an unconscious representation of agency from a conscious sense of agency, and this is what Dennett has called the “Hard Question” of consciousness.

## The hard question

The approach to the sense of agency used by traditional accounts such as the comparator model and the inference to mental state causation are only designed to answer one question about the sense of agency: how is a representation of one’s own agency elicited? This is a vitally important question in the study of the sense of agency, but to think it is the *only* question is to treat awareness as the end of the line of a computation, the dreaded Cartesian Theatre, and to deny the possibility of an unconscious representation of one’s own agency. In addition to this question, we also need to ask of accounts of agency what Dennett calls the “Hard Question” [not to be confused with any purported “Hard Problems” (Chalmers, 2003)^3^]: after the representation of own agency is elicited by one or other of these mechanisms, well, then what happens (Dennett, 1991, p. 255)? What is the difference between a representation of my own agency of which I become aware and one that languishes forever in the apparent irrelevance of unconsciousness?

The analogy employed above of “looking” for the sense of agency suggests one possible answer. Perhaps an unconscious representation of agency becomes a conscious representation when the subject’s attention is directed to it? In the following section I consider and discuss this possibility. Having found this wanting, I consider a further possibility, that the answer to the Hard Question is that the representation enters into the mechanisms required for verbal report. I argue that this answer is also unsatisfactory, as it is inconsistent with behavioral evidence of a sense of agency in non-verbal animals. These first two options are Functionalist theories. They propose that consciousness is playing a certain role in cognition. Although these two specific proposals seem to fail on empirical grounds it is important to note that other Functionalist theories, notably those that identify consciousness with multiple functional roles remain open. Finally, I propose a radical alternative suggesting that the answer to the Hard Question is to be found not in the uses to which representations are put within a cognitive system, but in the nature of the representations themselves. Regardless of which of the two research agendas individuals chose to pursue, it is clear that we do not have an answer to the Hard Question for the sense of agency nor do we spend enough time thinking about it.

## Attention

One potential answer to the Hard Question is *attention*. Such an answer is suggested by well-known cases of inattentional blindness, where subjects fail to see perfectly obvious stimuli (like a woman in a gorilla suit) simply because their attention is directed elsewhere (Mack and Rock, 1998). More specifically, let us hypothesize that the difference between an unconscious representation of agency and the conscious sense of agency is that the conscious representation is attended to. If this is true then we would have a clear research agenda: understand how and why a representation of agency is selected or not selected for attention and understand the mechanisms of attention.

Such a view has not been developed in detail for the sense of agency; indeed, I am suggesting here that consideration of the Hard Question with respect to the sense of agency has been neglected almost entirely. Notwithstanding, attention based accounts of consciousness do have some currency in the explanation of perceptual consciousness. Prinz (2000, 2012), for example, advocates such a view. Unfortunately evidence is mounting that attention is not a good answer to the Hard Question, at least not on its own, as attention is not sufficient for consciousness. That is, subjects can attend to things of which they are not conscious. Here I discuss one well-studied example.

**Figure 1:**
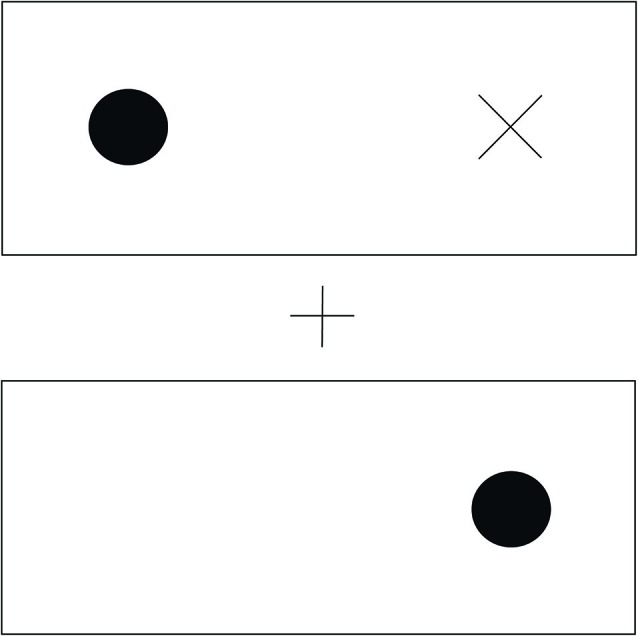
**The spatial layout of the stimuli.** In the center we see a fixation cross, above and below are two rectangles. A cue appears at the x and disappears, followed by a target at one of the two circles. The subject’s task is to indicate the color of the target. Subjects respond faster to cues appearing within the same shape as the cue, even though they are as far from the cue as the target in the other shape.

Norman et al. (2013) have provided compelling evidence that subjects can visually attend to objects, namely two-dimensional shapes, even when they cannot consciously see those objects. They start from prior observations of the effects of taking two-dimensional shapes as the objects of attention in color discrimination tasks. In these tasks, subjects are asked to indicate with a button press the color of a circle. Before the target colored circle appears a supraliminal spatial cue is presented. In the trials of interest here the, cue appears some distance from where the target circle will ultimately appear. However, it may appear in the same shape as the cue or a different shape. See Figure 1 for an example layout.

When the target appears in the same object as the cue, response times are facilitated (Egly et al., 1994). Norman et al. take this as characteristic of attention to such shapes.

Norman et al. repeated this experiment, but made the shapes invisible. They presented on a screen an array of Gabor patches whose orientation rapidly alternated between vertical and horizontal. Within the array rectangles were defined by Gabor patches flickering out of phase with the remainder of the array (Norman et al., 2013, p. 838). When the background patches were vertical, those defining the rectangle were horizontal, and vice versa. Observing the array subjects reported seeing flickering Gabor patches, but were unable to see the rectangles. Indeed, subjects were no better than chance when asked to guess whether or not such flickering displays contained rectangles (Norman et al., 2013, p. 840). Despite the invisibility of the shapes there was a facilitation effect in the color discrimination task characteristic of attention being directed at the shapes. That is, subjects were faster at responding to targets which appeared in the same shape as the cue, than for targets which appeared the same distance from the cue but in a different shape (Norman et al., 2013, p. 839).

In this study we see an effect characteristic of attention being directed at an object, despite the object being invisible. This demonstrates that subjects can attend to shapes of which they are not conscious. In general, this also suggests that attention is not sufficient for consciousness. Without a reason to think that the sense of agency will be an exception to this, it seems unlikely that attention will answer the Hard Question for the sense of agency.

## Reportability

Often we take it that we can be confident that a subject experiences something if they are able to verbally report it. Although such reports are susceptible to a variety of introspective omissions and commissions (Dennett, 1991, p. 96; Schwitzgebel, 2008), in practise, verbal reports (especially questionnaire responses) are very often treated as the best way to operationalize experience. Indeed the theories of the sense of agency introduced above are built on studies using questionnaires to ask subjects to report their experiences of agency. At the heart of this approach lies an intuition that, however imperfectly, we are able to talk about those things that we experience, but not those things that reside in our unconscious minds. This intuition suggests an approach to the Hard Question: perhaps the difference between conscious and unconscious representations is just that conscious representations are available for report. Although such an approach would be highly controversial (Block, 2007), there is no approach to the Hard Question that is not controversial, and this proposal remains live.

That said, we do have strong reason to doubt that it is reportability that distinguishes conscious and unconscious representations of own agency, as there are many non-verbal animals that display evidence of experiencing a sense of agency. This suggests that being available for verbal report is not necessary for a conscious sense of agency.

Good evidence for this comes from the mirror self-recognition test. This test, first proposed by Gallup (1970), involves marking an animal surreptitiously (usually when anesthetized) with a non-irritating, odorless dye on a part of the animal’s body that cannot be seen without a mirror (such as the forehead). An animal is deemed to pass the mirror self recognition test if there is a significant increase in mark directed behavior coincident with the animal observing itself in the mirror (Gallup, 1970, p. 87). Such behavior indicates that the animal has recognized itself in the mirror as it uses the mirror to direct actions towards itself. A sense of agency is needed to pass such tests. To learn to recognize oneself in a mirror one needs to realize that the actions one sees in the mirror are equivalent to the actions one is currently performing (Povinelli, 2001, p. 855). In order to recognize oneself in a mirror, then, one needs to know (amongst many other things) what action one is performing. This is a function of the sense of agency (Povinelli and Cant, 1995). As such, passing the test is good evidence for a sense of agency.

Where this creates a problem for using reportability as an answer to the Hard Question is in the fact that many non-verbal animals pass the mirror self-recognition test. This includes chimpanzees (Gallup, 1970), orang-utans, human raised gorillas (Povinelli and Cant, 1995), bottlenose dolphins (Marten and Psarakos, 1994) and European magpies (Prior et al., 2008). These animals thus show evidence of experiencing a conscious sense of agency. As such, verbal report does not seem necessary for consciousness, and thus investigating how unconscious representations of agency become available for verbal report is a non-starter as a solution to the Hard Question.

The solutions considered so far to the Hard Question are Functionalist theories. They posit that for a representation to be conscious is for it to be used a certain way, say be being attended to or by being made available for report. On such views it is *use* which constitutes consciousness. Whilst the two options considered here do seem like non-starters, there are other Functionalist theories available. Other accounts, such as Dennett (1991) multiple drafts model Dennett (1991) or Baars (1988) global workspace Baars (1988), suggest that consciousness is not a single use within a cognitive system, but rather a conglomeration of many uses and these options remain live. My point here is not to solve the problem of what distinguishes conscious and unconscious representations, but merely to suggest that in sense of agency research this is a problem we should spend more time on. Next, I turn to a theoretical basis for approaching the Hard Question that offers a fundamentally different kind of solution to the options considered so far.

## Vehicle theories

Vehicle theories of consciousness answer the Hard Question in a rather different way. The key issue we are getting at is: what is the difference between an unconscious and a conscious representation of own agency? The proposals considered thus far have followed Dennett in hypothesizing that this difference is a difference between how unconscious and conscious representations are processed (e.g., are they subject to attention or made available for verbal report). In other words the difference is a matter of what is done with the representation. Such approaches are Functionalist theories in that they consider the particular use of a representation within a cognitive system to constitute that representation’s being conscious.

Vehicle theories, in contrast, hypothesize that the difference between conscious and unconscious representations is not how they are processed, but in the nature of the representation itself (O’Brien and Opie, 1999, p. 128). The nature of conscious vehicles of representation (also known as representation bearers) is hypothesized to be different to the nature of unconscious representing vehicles. On such views consciousness is a way of representing the world using different kinds of vehicle than those used by unconscious representations. On this kind of view the answer to the Hard Question is not “and then some additional processing occurs” but rather, “and then the vehicle of representation is changed from one form to another”.

O’Brien and Opie propose a general answer to this question making use of distinctions in kinds of representing vehicles offered by Dennett (1982). In particular they focus on a distinction between “explicit” representations which are: “physically distinct objects, each possessed of a single semantic value” (O’Brien and Opie, 1999, p. 133) and “potentially explicit” and “tacit”^4^ representations which are to be understood in terms of a computational system’s *capacity* to make certain information explicit in the above sense. In general, O’Brien and Opie hypothesize that we are conscious of all and only things that are represented in an explicit form. All unconscious representations would then take the form of potentially explicit or tacit representations.

According to this version of a Vehicle Theory, a conscious sense of agency would be an explicit representation of own agency. That is, a discrete vehicle, such as a stable pattern of activity across a layer of neurons (O’Brien and Opie, 1999, p. 138), with that content. An unconscious representation of agency would not be a discrete vehicle, but a disposition in the cognitive system to produce such a representation. To allow for unconscious representations of own agency on such a view, the output of the comparator model or Wegner et al. inference needs to be reconceived. It is not an explicit representation of own agency, but rather a change in the dispositions of a computational system to produce such a representation.

If such an approach is correct then we have a new way to approach the Hard Question for the sense of agency. How is the output of the comparator model, or whichever account we ultimately agree on, made explicit? Why is it the case that it is sometimes not made explicit? Is this a matter of the subject metaphorically “looking for” it, if so, how would that be understood more literally?

The benefit of taking this approach is that it offers a new kind of answer to the Hard Question by offering a new conception of what properties of a computational system distinguish conscious from unconscious representations. With this reconceptualization we can deploy O’Brien and Opie’s hypothesis for the sense of agency and answer the Hard Question in a way that doesn’t seem to be falsified like the other answers considered here. In addition, a research agenda is set: why and how is a representation of own agency sometimes made explicit? Of course this question has not yet been answered. Indeed whichever form of the Hard Question we prefer it is clear that we have not yet answered it, although there seem to be two promising avenues to approach it. And so I implore us, as a community to ask of ourselves, now that we have made progress in understanding how a representation of own agency is elicited, *then what happens?*

## Conclusion

In this paper I have argued that in order to explain the sense of agency we need to move beyond proximal etiological explanations and consider the Hard Question. Although such accounts, including the comparator model and the inference to apparent mental sate causation, are powerful so far as they go, they fail to distinguish between conscious and unconscious representations of own agency. As a consideration of the occasionality problem suggests, not only is this a real distinction, but such representations can play very different roles in cognition. Finally, I have suggested that there are ways we can approach the Hard Question, and although some of the specifics of certain particular approaches might seem like non-starters on empirical grounds it should be clear that there are alternative approaches, both Functionalist and vehicle, available and specific questions to ask. Now what happens?

## Conflict of interest statement

The author declares that the research was conducted in the absence of any commercial or financial relationships that could be construed as a potential conflict of interest.

## Footnotes

^1^As a rough guide 1 degree is approximately the angle subtended by a point either side of your thumb nail held at arms length.

^2^Now of course in the normal case our eyes saccade constantly allowing us to build a much more detailed visual representation than is possible from staring at a fixation point making the area of clear vision significantly larger than the 2 to 3° observable in a fixation task. This doesn’t affect the central Dennettian claim that periphery is not clear and colored in the way that we would typically assume. Nor do we typically reflect on howmuch moving our eyes is necessary for seeing the way we do.

^3^The supposed “Hard Problem” of consciousness is the problem of explaining how mental and physical states give rise to conscious experience, given that it seems that no explanation in terms of the structure or function of mental states is sufficient to explain this (Chalmers, 2003). This is a problem closely tied with mysterian and dualistic approaches to consciousness. In contrast the “Hard Question” is a question within the materialist tradition, which works from arguments that a structural or functional explanation of consciousness is possible in principle, and asks what is the difference between a conscious and unconscious mental representation.
